# Cervical esophageal adenocarcinoma of intestinal type in ectopic gastric mucosa

**DOI:** 10.1002/deo2.141

**Published:** 2022-06-18

**Authors:** Ryosuke Ikeda, Kingo Hirasawa, Yuichiro Ozeki, Atsushi Sawada, Masafumi Nishio, Takehide Fukuchi, Chiko Sato, Shin Maeda

**Affiliations:** ^1^ Endoscopy Division Yokohama City University Medical Center Kanagawa Japan; ^2^ Department of Gastroenterology Yokohama City University Graduate School of Medicine Kanagawa Japan

**Keywords:** cervical esophageal adenocarcinoma, ectopic gastric mucosa, endoscopic submucosal dissection, intestinal metaplasia, intestinal‐type

## Abstract

A 45‐year‐old man underwent esophagogastroduodenoscopy because of symptoms of laryngopharyngeal discomfort. We found a protruded reddish lesion adjacent to the ectopic gastric mucosa (EGM) in the cervical esophagus, and a biopsy revealed that it was a tubular adenocarcinoma. We diagnosed the patient with intramucosal cancer and performed endoscopic submucosal dissection. Esophageal endoscopic submucosal dissection was performed under general anesthesia using a conventional procedure. The resected tumor measured 23 × 14 mm and was adjacent to the EGM. Histologically, the tumor cells showed moderately well‐differentiated adenocarcinoma confined to the muscularis mucosa with no lymphovascular infiltration. Immunohistochemically, the tumor cells were positive for intestinal markers, namely MUC2 and CD10, and negative for gastric markers, namely MUC5AC and MUC6. The patient had no post‐endoscopy submucosal dissection stenosis and remained disease‐free without local recurrence. EGM of the cervical esophagus develops from the columnar epithelium during embryonic development. There are few reports on endoscopic submucosal dissection for mucosal cancer. Of these, immunostaining was performed in three cases. All were positive for MUC5AC and MUC6 and negative for MUC2 and CD10. Usually, EGM shows gastric type epithelium, but occasional cases with intestinal metaplasia, which show positivity for MUC2 and CD10, have been reported. Therefore, we consider this to be an extremely rare case of esophageal adenocarcinoma arising from intestinal metaplasia within the EGM.

## INTRODUCTION

Ectopic gastric mucosa (EGM) of the cervical esophagus develops from columnar epithelium during the embryonic period,^1^ with a frequency of approximately 11%.^2^ Esophageal adenocarcinoma, which develops from EGM, is extremely rare. We report a case of cervical esophageal adenocarcinoma of the intestinal type that was resected by endoscopic submucosal dissection (ESD).

## CASE REPORT

**FIGURE 1:**
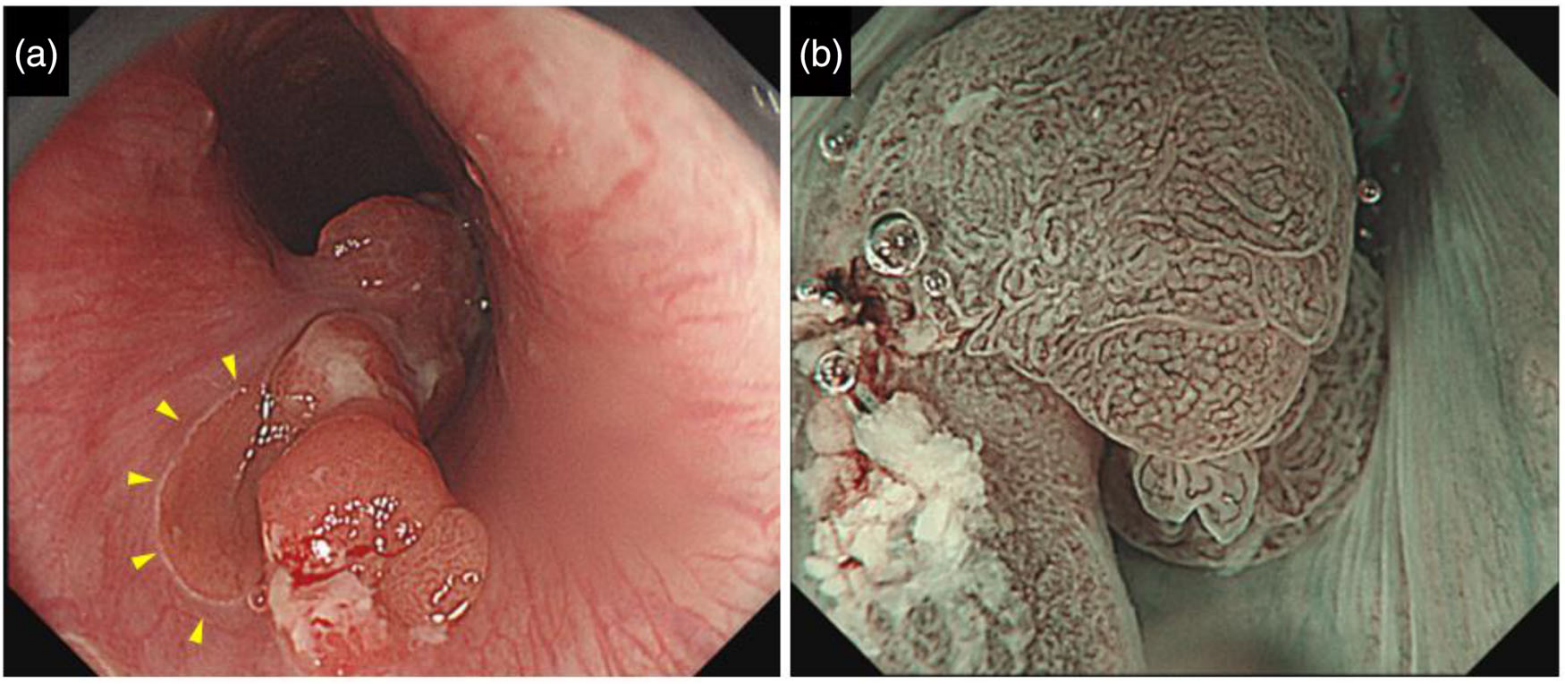
(a) White‐light imaging revealed a protruded reddish lesion in the cervical esophagus. The lesion was adjacent to the ectopic gastric mucosa. (arrowheads). (b) Narrow band imaging with magnifying endoscopy showed a clear demarcation line of the tumor, consistent with a reddish lesion in the white‐light imaging. The surface of the tumor displayed irregular glandular and villous structures with irregular microvessels

A 45‐year‐old man underwent esophagogastroduodenoscopy (EGD) because of symptoms of laryngopharyngeal discomfort. We found a protruded reddish lesion adjacent to the EGM in the cervical esophagus (oral verge, 18 cm). The surface of the tumor showed a glandular and villous‐like structure, and narrow‐band imaging endoscopy showed an irregular microsurface and microvessel pattern (Figure 1). We endoscopically diagnosed the lesion as an adenocarcinoma of the EGM. The lesion was biopsied and diagnosed as tubular adenocarcinoma. Since the tumor showed no findings of submucosal invasion and we confirmed that there was no lymph node metastasis by performing computed tomography, we considered it to be intramucosal cancer and performed ESD.

**FIGURE 2:**
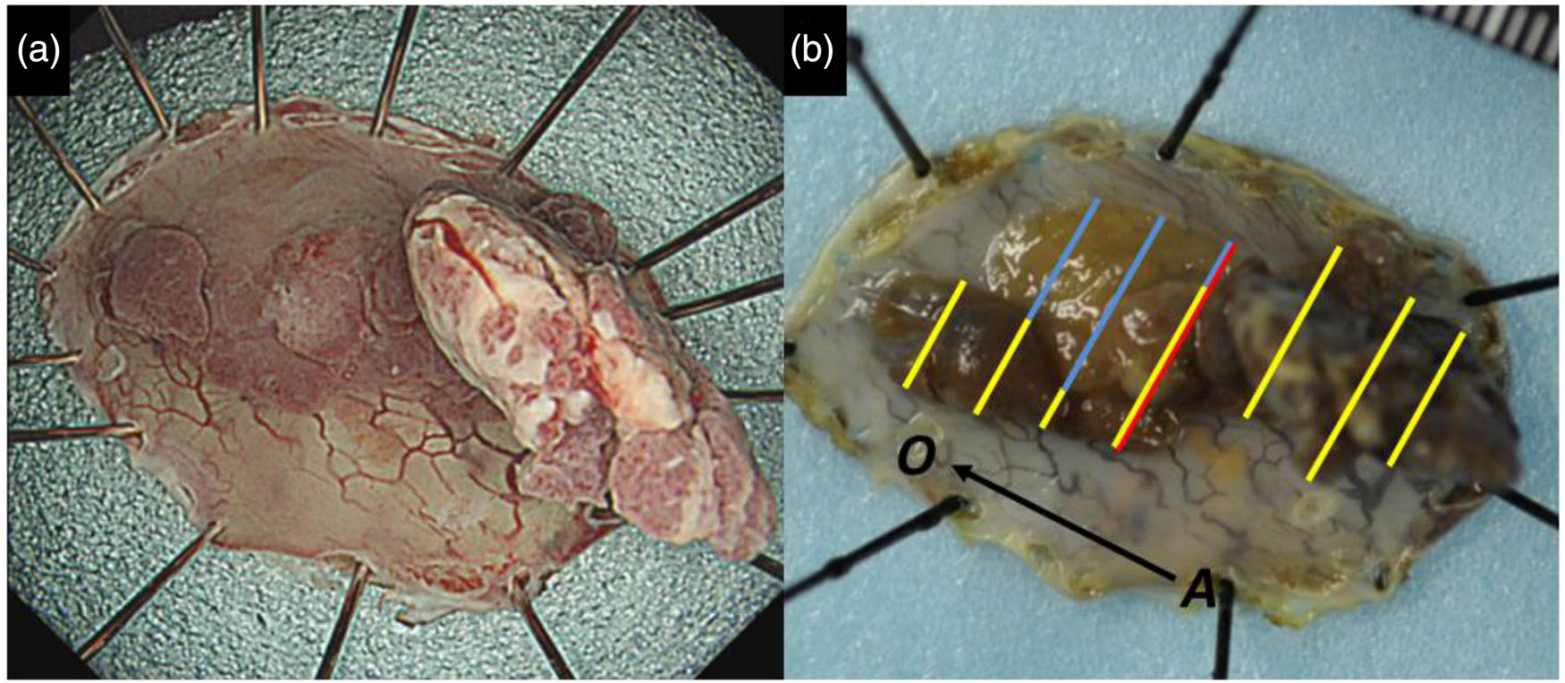
(a) The resected specimen showed a flat elevated lesion with a protruded structure partially adjacent to the ectopic gastric mucosa. (b) Mapping of the endoscopic submucosal dissection specimen based on histology. The resected specimen was cut into 2‐mm thick sections vertically to the oral‐anal (OA) line. Adenocarcinoma and the ectopic gastric mucosa were distributed along the yellow and blue lines, respectively. The pathological findings of the red line are shown in Figure 3

**FIGURE 3:**
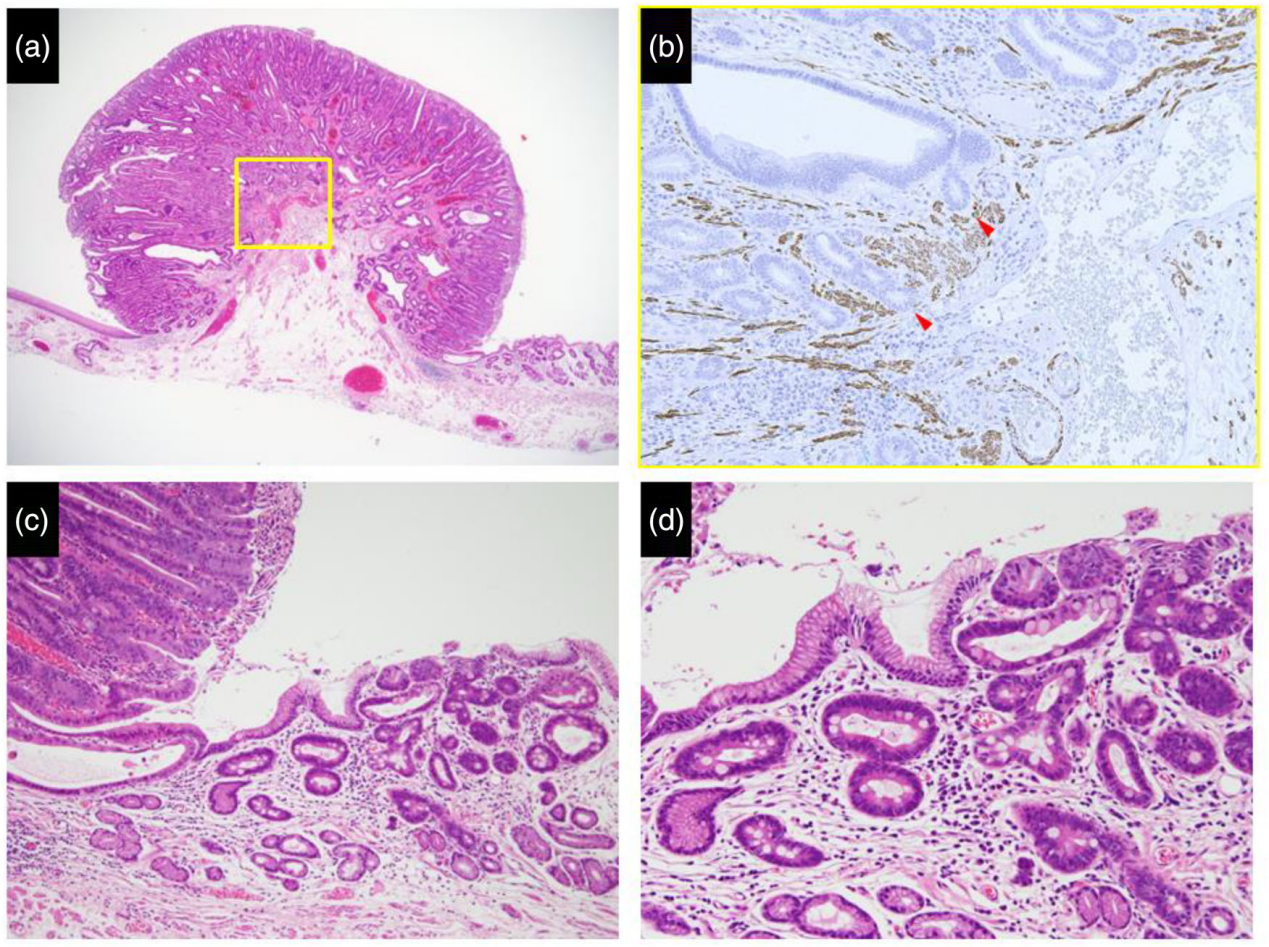
(a) Histologically, the protruded lesion, which contained abnormal glandular structures, was adjacent to the ectopic gastric mucosa. (b) This is the desmin staining of the yellow square in Figure 3a. The tumor partially invaded the muscularis mucosa (arrow heads). (c) Ectopic gastric mucosa was found adjacent to the tumor. (d) The epithelium of the ectopic gastric mucosa showed focal intestinal metaplasia

**FIGURE 4:**
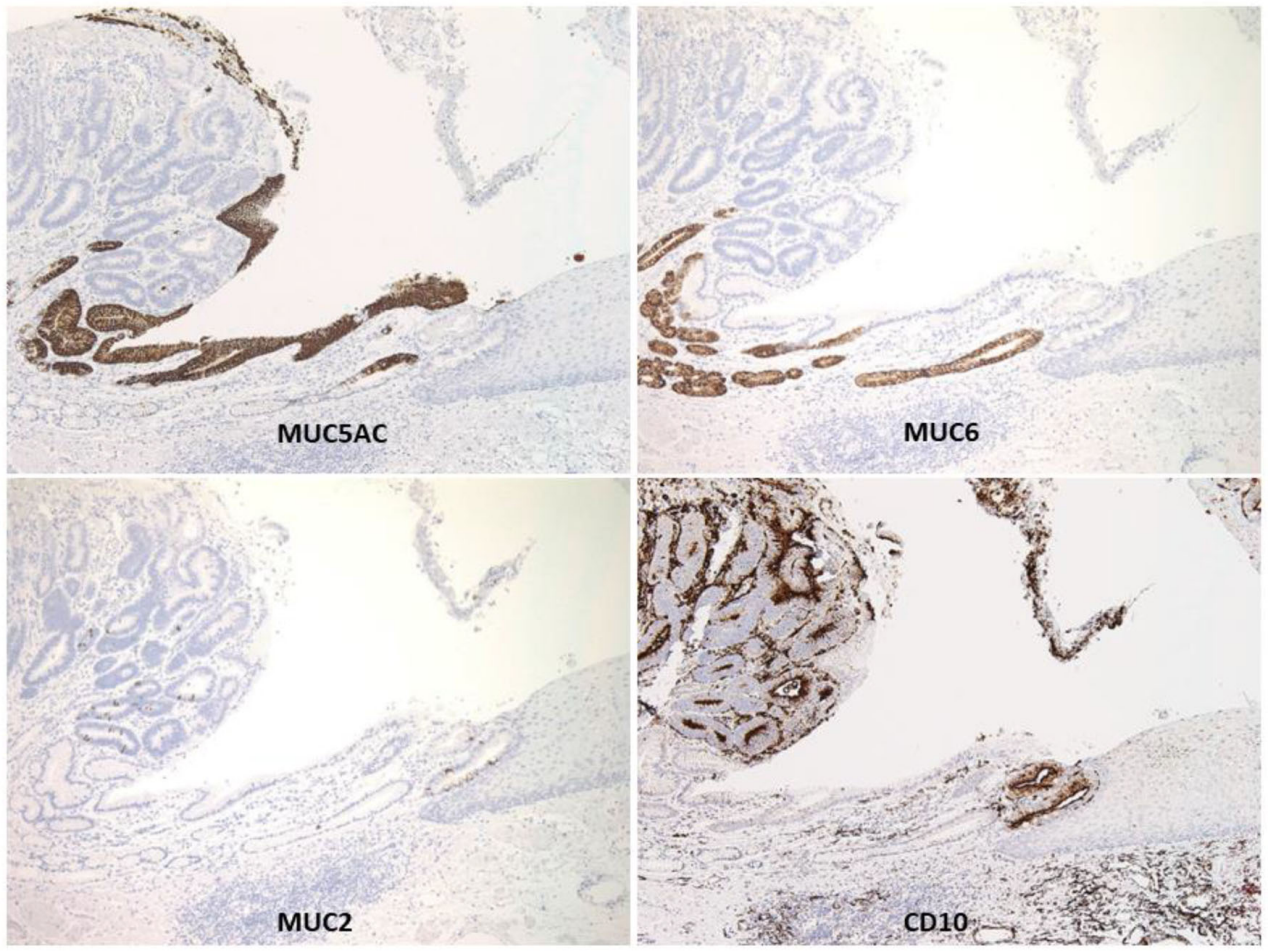
Immunohistochemical staining of the resected specimen. Abnormal glandular structures were negative for MUC5AC and MUC6 but showed CD10 positivity and focal MUC2 positivity. The ectopic gastric mucosa was positive not only for MUC5AC and MUC6 but also for CD10 and focal MUC2

Esophageal ESD was performed under general anesthesia using a conventional procedure. We used an endoscope that provides narrow‐band imaging with magnifying endoscopy (GIF‐H290Z; Olympus Medical Systems, Co., Tokyo, Japan) to accurately confirm the tumor margins, and ESD was performed using a single‐channel endoscope with a water jet (GIF‐Q260J; Olympus) with a high‐frequency power supply unit (VIO300D; ERBE, Tübingen, Germany) for electrocoagulation. After marking around the tumor, we started a mucosal incision using a dual knife (Olympus Medical Systems, Co.) and performed submucosal dissection using an insulated‐tip knife nano (Olympus Medical Systems, Co.) and a dual knife (Figure 2). After resection, we injected a steroid (triamcinolone acetonide; Kenacort; Bristol Myers Squibb, USA) into the artificial ulcer to prevent postoperative stenosis. The resected specimen measured 40 × 24 mm and contained a macroscopically measured 23 × 14 mm tumor adjacent to the EGM. Histologically, the tumor cells showed moderately well‐differentiated adenocarcinoma confined to the muscularis mucosa (Figure 3). No lymphovascular infiltrations were observed. Immunohistochemically, the tumor cells were focally positive for mucin (MUC) 2 and CD10, and negative for MUC5AC and MUC6, suggesting that the tumor was of the intestinal type, not gastric type. The surrounding non‐cancerous area showed focal MUC2 positivity and CD10‐positive intestinal mucosa similar to the cancerous lesion. MUC5AC‐ and MUC6‐positive mucosa around the lesion were considered to be the EGM (Figure 4). At present, there is no strong evidence of the prognosis and lymph node metastasis in the superficial esophageal adenocarcinoma of the EGM. Therefore, we decided to follow up with the patient under strict examination without additional treatment based on the Guidelines for Diagnosis and Treatment of Carcinoma of the Esophagus. After 6 months, the patient had no post‐ESD stenosis and remained disease‐free without local recurrence. We will consider a follow‐up schedule for EGD and computed tomography every 6 months.

## DISCUSSION

Esophageal adenocarcinoma developing within the EGM is extremely rare, and there have only been a few case reports of ESD performed for mucosal cancer.^3^, ^4^, ^5^, ^6^ In these reports, there were three cases in which immunostaining was performed, and all were positive for gastric MUC markers MUC5AC and MUC6 and negative for intestinal markers MUC2 and CD10. EGM is thought to be a remnant columnar epithelium that should have transformed into squamous epithelium during the embryonic period but instead persisted. Usually, the EGM contains gastric type epithelium, but a few cases showing intestinal metaplasia have been reported.^7^

This case was positive for immunostaining of the intestinal type not only in the tumor but also in the surrounding epithelium. Therefore, we believe that the tumor developed from intestinal metaplasia. There are no reports that directly mention the cause of intestinal metaplasia in EGM. Although the involvement of bile acids has been reported in Barrett's epithelium,^8^ EGM is unlikely to be affected by bile acids due to its anatomical location. Regarding *Helicobacter pylori* infection, it has been reported that *H. pylori* were not found in the EGM of patients with *H. pylori*‐negative atrophic gastritis.^9^ In this case, no endoscopic features of *H. pylori* infection were found (data not shown), and other factors may be involved in intestinal metaplasia of the EGM. There are no reports of esophageal adenocarcinoma arising from intestinal metaplasia within the EGM; therefore, this case is extremely rare and gives valuable insight into the development of esophageal adenocarcinoma of the intestinal type.

## CONFLICTS OF INTEREST

The authors declare that they have no conflict of interest.

## FUNDING INFORMATION

None.
